# Developing more generalizable prediction models from pooled studies and large clustered data sets

**DOI:** 10.1002/sim.8981

**Published:** 2021-05-05

**Authors:** Valentijn M. T. de Jong, Karel G. M. Moons, Marinus J. C. Eijkemans, Richard D. Riley, Thomas P. A. Debray

**Affiliations:** ^1^ Julius Center for Health Sciences and Primary Care, University Medical Center Utrecht Utrecht University Utrecht The Netherlands; ^2^ Cochrane Netherlands, Julius Center for Health Sciences and Primary Care, University Medical Center Utrecht Utrecht University Utrecht The Netherlands; ^3^ Centre for Prognosis Research, School of Medicine Keele University Staffordshire UK

**Keywords:** heterogeneity, individual participant data, internal‐external cross‐validation, prediction

## Abstract

Prediction models often yield inaccurate predictions for new individuals. Large data sets from pooled studies or electronic healthcare records may alleviate this with an increased sample size and variability in sample characteristics. However, existing strategies for prediction model development generally do not account for heterogeneity in predictor‐outcome associations between different settings and populations. This limits the generalizability of developed models (even from large, combined, clustered data sets) and necessitates local revisions. We aim to develop methodology for producing prediction models that require less tailoring to different settings and populations. We adopt internal‐external cross‐validation to assess and reduce heterogeneity in models' predictive performance during the development. We propose a predictor selection algorithm that optimizes the (weighted) average performance while minimizing its variability across the hold‐out clusters (or studies). Predictors are added iteratively until the estimated generalizability is optimized. We illustrate this by developing a model for predicting the risk of atrial fibrillation and updating an existing one for diagnosing deep vein thrombosis, using individual participant data from 20 cohorts (N = 10 873) and 11 diagnostic studies (N = 10 014), respectively. Meta‐analysis of calibration and discrimination performance in each hold‐out cluster shows that trade‐offs between average and heterogeneity of performance occurred. Our methodology enables the assessment of heterogeneity of prediction model performance during model development in multiple or clustered data sets, thereby informing researchers on predictor selection to improve the generalizability to different settings and populations, and reduce the need for model tailoring. Our methodology has been implemented in the R package metamisc.

## 1 BACKGROUND

Large combined clustered data sets are increasingly available, for example in so‐called individual participant data meta‐analyses (IPD‐MA) projects (where the data are clustered by study) and in studies using large scale electronic healthcare records (where the data are clustered by region, hospital, practice, etc).^1^ Such data sets are frequently used to develop prediction models, to predict a current health status to aid in diagnosis or a future health outcome to provide a prognosis which may inform clinical decision making.^2^, ^3^, ^4^ Well‐known examples are PHASES,^5^ INTERCHEST,^6^ S_2_TOP‐BLEED,^7^ and EuroSCORE,^8^ all of which were developed using data from multiple centers or studies. Unfortunately, prediction model studies that are based on IPD‐MA or electronic healthcare records (EHR) rarely account for the potential of between‐cluster heterogeneity (eg, EuroSCORE^8^).^9^, ^10^ Sometimes, parameters that capture the baseline risk are stratified by cluster (eg, INTERCHEST^6^), but then usually no guidance is provided on how to use the prediction model in new patients.

Although random effects models are generally recommended for dealing with the presence of clustering and heterogeneity of predictor effects, their implementation during prediction model development hampers the applicability of the estimated regression coefficients. In particular, random effects modeling does not indicate which parameter values (for the random intercept and predictor coefficients) should be used when the model is applied in new settings and populations. Typically, a single value (eg, the mean) is used for these parameters when making predictions.

When new observations or whole new clusters become available, the common and random effects of the model may be updated as new data become available, through the use of dynamic random effects methods.^11^ This can improve the predictive performance of the prediction model in a validation setting. However, when the model is used to provide a prediction for an individual that is not part of a known cluster, there is no information that can be used to update the model, which eliminates the option of applying a dynamic method.

In general, a developed prediction model cannot generate accurate predictions in new patients when the true value for its parameters (eg, the intercept term) varies across the targeted settings and populations, especially when the true value of certain parameters is zero or has a reversed sign in some clusters. This heterogeneity may arise from differences in observed and unobserved patient characteristics, differences in patients' treatment and management strategies, differences in predictor and outcome definitions and differences in measurement methods across clusters. These differences may be temporal, geographic, or domain in nature. A temporal validation, where the validation study is typically performed with a similar study design and protocol by the same authors but in individuals selected from a later time period as the development study, is considered to be the weakest form of external validation. Whereas small differences in the study population or quality of care in a geographic validation may be present, in a domain validation the target population or targeted outcome can be different. For instance, in a domain validation one may investigate whether a prediction model developed in primary care is transportable to secondary or tertiary care.^12^

The impact of heterogeneity in predictive associations (ie, the effects of predictors in the included model) has been well documented in the literature (Table 1).^13^, ^14^ Many developed prediction models perform poorer than anticipated and require local revisions prior to implementation.^15^ These revisions may involve a simple intercept update, a recalibration of the linear predictor (ie, rescale all regression coefficients by a single value), the re‐estimation of all the individual regression coefficients, or even the inclusion of new predictors.^12^, ^16^, ^17^, ^18^ Unfortunately, revisions are rarely generalizable to other settings and populations; several reviews have found that prediction model performance substantially varies across validation studies.^19^, ^20^ Therefore, such revisions, including recalibration and predictor selection, are preferably performed during prediction model development. We consider situations where it is not possible (or very difficult) to define a priori the potential source(s) of between‐cluster heterogeneity, such as temporal calibration drift.^21^, ^22^ We aimed to develop methodology that is universally applicable, when causes of poor transportability cannot be directly addressed a priori.

**TABLE 1:** Types of heterogeneity

• Within‐cluster heterogeneity of (measured and unmeasured) covariate values. This heterogeneity is necessary to allow for individual risk prediction
• Between‐cluster heterogeneity in the distribution (eg, mean and SD) of predictor values. This heterogeneity may appear when clusters represent different geographic regions, countries or studies, etc
• Between‐cluster heterogeneity in baseline risk (eg, outcome incidence). This is often related to between‐cluster heterogeneity in eligibility criteria (eg, randomized trial versus observational study or between settings with differences in patient management), or to between‐cluster heterogeneity in unmeasured predictor values
• Between‐cluster heterogeneity of predictor‐outcome associations (and thus in the estimated model coefficients). These differences may, for instance, appear when combining data from studies with differences in patient management, variable and outcome definitions, etc
• Between‐cluster heterogeneity of prediction model performance. The “true” performance of a prediction model may vary between clusters due to differences in their underlying predictor‐outcome associations, or due to differences in their distribution of (measured or unmeasured) predictor values^13^, ^14^, ^31^

The identification of between‐cluster heterogeneity is not possible when data are available from only a single setting or (sub)population. For this reason, the use of clustered data during prediction model development and its subsequent validation offers a critical opportunity to inspect whether this heterogeneity would actually be a concern when the model would be implemented in clinical practice.^9^, ^10^, ^13^, ^14^, ^23^, ^24^, ^25^, ^26^, ^27^, ^28^ However, actually resolving the presence of between‐cluster heterogeneity (and thus ensuring model predictions are accurate for all clusters) remains a difficult challenge for which limited guidance is available.^29^ For this reason, we here explore an alternative approach that aims to reduce this between‐cluster heterogeneity in prediction model performance and minimize the need for estimating setting‐specific model parameters, to thereby improve its generalizability.

Recently, internal‐external cross‐validation (IECV) has been introduced to assess the presence of heterogeneity of a model's performance during its development.^10^, ^23^, ^25^ IECV is a special case of cross‐validation; available data are split nonrandomly in a natural manner by iteratively taking each cluster (or study) as a hold‐out sample. In each iteration, a model is developed on the retained clusters, and then the model is tested in the hold‐out cluster. A key advantage of this is that it allows the transportability (ie, the generalizability to other populations and settings) of the model to be assessed multiple times.

In this article, we will first revisit the IECV framework for assessment of model performance in large clustered data sets (Section 2). For the highlights of this article, see Table 2. We then extend the IECV framework to inform predictor selection during prediction model development in Section 3, in order to identify and reduce their impact on the model's performance within and across clusters in the large combined data set. We then apply the methods in our motivating examples in Sections 4 and 5. Finally, we provide a discussion in Section 6. Our methodology can be applied using the metapred function in the R package metamisc.^30^

**TABLE 2:** Highlights

• Many studies have shown that overfitting can lead to poor performance when predicting the outcome in new participants, but also that this can be resolved by utilizing large data sets^35^, ^36^, ^37^
• Prediction models may still perform poorly even when overfitting is not an issue. This situation can occur when predictor effects do not transport well across different settings.^10^, ^38^, ^39^ SIECV aims to address this issue, by incorporating this transportability in the model building process
• SIECV is designed for situations where it is difficult to locally update model predictions. It aims to yield a global model with stable performance across the relevant settings and (sub)populations, and may therefore have suboptimal performance within (some of the) individual clusters, particularly when they differ much from one another
• SIECV can help to find an appropriate balance between a model's internal and external validity, and to limit the need for local revisions (by avoiding heterogeneity in predictor‐outcome associations)
• SIECV does not address miscalibration‐in‐the‐large. This can be resolved using a relatively simple update of the model's intercept term, and will often remain necessary.^18^, ^40^ Finally, access to clustered data allows one to estimate multiple intercept terms, and thus to ascertain under which conditions an intercept update may be necessary^9^, ^13^

## 2 INTERNAL‐EXTERNAL CROSS‐VALIDATION FOR MODEL VALIDATION

Resampling procedures allow the optimal use of the available data, as all data can be used for model development and subsequent evaluation. Traditionally in cross‐validation procedures, the data are iteratively split into a development and validation set by randomly sampling without replacement. In each iteration, a model is estimated on the development sample and predictions are made for the random validation sample. The performance of these predictions in the validation samples is then averaged across iterations, thereby giving an estimate of the reproducibility of model performance.

When data are clustered across different studies or settings, traditional resampling procedures that do not account for clustering cannot directly be applied.^32^ For this reason several extensions have been proposed that preserve the clustering within and the heterogeneity across the generated samples. In the so‐called internal‐external cross‐validation approach, the data are split by cluster, which may represent the studies from an IPD‐MA or the centers in data from EHR.^23^, ^25^, ^26^ A model is then iteratively fit in K‐1 clusters (Section 2.1) and its corresponding performance model performance is calculated in the remaining cluster (Section 2.2). This is repeated K times, so that, provided that sufficient data are available in the development and validation clusters, a performance estimate and its SE is available for each of the clusters. Thus, IECV is cross‐validation where the hold‐out samples are nonrandom, in the presence of between‐cluster heterogeneity. IECV therefore allows the study of a developed model's potential transportability multiple times. Note that if all patients are exchangeable across clusters, IECV corresponds to the traditional cross‐validation and assesses model reproducibility (rather than transportability).^10^

In contrast to traditional cross‐validation, estimates of the performance in the hold‐out samples cannot simply be averaged, as the variation within and across clusters needs to be taken into account. This can be achieved by adopting a (fixed effect or random effects) meta‐analysis of the performance estimates (Section 2.2),^33^ or by weighting the performance estimates by the number of events in each cluster.^34^ As the data are split nonrandomly, this allows the transportability (ie, the generalizability to other populations and settings) of the model to be assessed.

### 2.1 Model fitting

The development phase of IECV may involve a one‐stage or a two‐stage IPD‐MA approach. In the two‐stage approach, the prediction model is fitted separately in each cluster. The model coefficients estimated in each of the development K‐1 clusters are then combined using standard meta‐analysis techniques. In the one‐stage approach, a generalized linear model (GLM) is estimated in each of the *K* development samples consisting of *K* − 1 clusters. This model may account for clustering by including random intercepts and/or slopes for within‐cluster covariates.^13^, ^14^, ^29^, ^34^ A disadvantage of the one‐stage approach in IECV is that the data from each cluster needs to be used K‐1 times to fit a model in the one‐stage approach. On the other hand, in the two‐stage IECV approach the data from each cluster only needs to be used for model fitting once, as the second stage comprises meta‐analysis of different combinations of coefficients and their SEs. The two‐stage approach may therefore substantially reduce the necessary computational performance time. However, the two‐stage approach may not be feasible when clusters are relatively small, as parameters then become difficult to estimate. For this reason, the two‐stage approach appears beneficial when most clusters (studies) in the meta‐analysis are not small, and we adopt this approach in our article.

Let *x*_*p*, *k*, *j*_ be the value of a prespecified predictor p, p = 1,…,P (or function thereof) measured in individual patients *j*, *j* = 1, … , *N* in cluster *k*, *k* = 1, … , *K*. Then their outcomes *y*_*k*, *j*_ may be modeled as follows:

(1) $$y_{k,j}=f(\alpha_{k}+\overset{P}{\underset{p=1}{\sum }}\beta_{p,k}x_{p,k,j}),$$

where *f*(…) is a link function, $\alpha_{k}$ is a cluster‐specific intercept and $\beta_{p,k}$ is a cluster‐specific coefficient. Here, we propose to estimate $\alpha_{k}$ and $\beta_{p,k}$ in each cluster separately. Subsequently, the estimates can be summarized using traditional meta‐analytic methods. We here use univariate random effects meta‐analysis, where each of the estimated coefficients are summarized separately:

(2) $${\hat{\beta }}_{p,(h)}^{\text{MA}}=\frac{\sum_{k\neq h}w_{p,k}{\hat{\beta }}_{p,k}}{\sum_{k\neq h}w_{p,k}},$$

where *w*_*p*, *k*_ is the weight attributed to ${\hat{\beta }}_{p,k}$ estimated in cluster *k*, and ${\hat{\beta }}_{p,(h)}^{\text{MA}}$ is the meta‐analytic estimate of the coefficient estimated on data from all clusters except hold‐out cluster *h*. In the random effects model the *w*_*p*, *k*_ are given by $\frac{1}{\mathrm{var}({\hat{\beta }}_{p,k})+\tau^{2}}$, where $\tau^{2}$ is the statistical heterogeneity estimate of the coefficient across clusters:

(3) $$\begin{array}{ll} {\hat{\beta }}_{p,k} & ∼𝒩\left(\beta_{p,k},\mathrm{var}\left({\hat{\beta }}_{p,k}\right)\right),\;\\ \beta_{p,k} & ∼𝒩\left(\beta_{p,(h)}^{\text{MA}},\tau_{p,(h)}^{2}\right). \end{array}$$

A confidence interval (CI) for ${\hat{\beta }}_{p,(h)}^{\text{MA}}$ is preferably constructed with the Hartung‐Knapp approach: ${\hat{\beta }}_{p,(h)}^{\text{MA}}\pm t_{Q-1,1-\alpha /2}\;\sqrt[{}]{var_{\text{HK}}({\hat{\beta }}_{p,(h)}^{\text{MA}})}$, where $t_{Q-1,1-\alpha /2}$ is the upper α/2 quantile of a *t*‐distribution with *Q* − 1 degrees of freedom, $var_{\text{HK}}({\hat{\beta }}_{p,(h)}^{\text{MA}})$ is a modified variance estimate and *Q* = *K* − 1 as one cluster is held out for validation.^41^, ^42^, ^43^, ^44^, ^45^ The extent of heterogeneity of a predictor effect can be explored by quantifying a prediction interval (PI), which estimates the interval of probable predictor effects in a new individual cluster, and can be calculated approximately as ${\hat{\beta }}_{p,(h)}^{\text{MA}}\pm t_{Q-2,1-\alpha /2}\;\sqrt[{}]{{\hat{\tau }}_{p,(h)}^{2}+var({\hat{\beta }}_{p,(h)}^{\text{MA}})}$.^46^, ^47^ A wide prediction interval for the predictor effect indicates that the predictor effect may be very different in a new cluster, which makes it unlikely that the predictor will improve the model's predictions for individuals in a new cluster.

The random effects meta‐analysis model is preferably estimated with REML or the Paule‐Mandel method.^48^, ^49^, ^50^, ^51^ When fewer than 10 clusters are included in the meta‐analysis, or when some clusters are small or the outcome is rare, the between‐cluster heterogeneity cannot be reliably estimated by any currently available method.^51^ The estimated coefficients could also be summarized using multivariate meta‐analysis methods,^33^, ^52^ which may be helpful in the presence of collinearity and missing parameter estimates. The necessary within‐cluster covariances can then directly be estimated from the IPD set at hand. However, usually univariate and multivariate meta‐analysis methods give very similar results when all of the parameter estimates of interest are available for all clusters, even when correlations are large.^53^ In IECV, all parameters can be estimated from the data hand, meaning that univariate meta‐analysis will usually suffice.

### 2.2 Assessing external model validity

In each iteration of the IECV, the developed model is validated in individuals from the hold‐out cluster by applying the model (as developed in the other clusters) using the observed predictor values of individuals. If the developed model contains random (or stratified) intercept terms or predictor effects, this also requires choices about which parameter values are to be used when applying the developed model.

It may be fruitful to select an intercept or predictor effect that best fits the validation cluster,^13^ provided that sufficient data are available for the validation cluster. Alternatively, given that the prediction model is mean centered and the overall frequency of the outcome is known for the population or cluster that the model is applied to, the appropriate intercept can be restored.^13^ When no such options are available, one may choose to apply the mean of the random intercept or predictor effects. Recent work shows that in terms of calibration the latter outperforms marginal models and models that ignore clustering during model development.^54^

When comparing the risk predictions for the hold‐out cluster with the observed outcomes, several performance measures such as the c‐statistic, calibration slope, calibration‐in‐the‐large and/or mean square error can be calculated.^10^, ^27^, ^55^ This process is repeated until each cluster has been used as a hold‐out cluster once, yielding a set of performance statistics for each IECV iteration. The corresponding estimates can then be pooled across the hold‐out clusters using random effects meta‐analysis methods, though some statistics and their SEs may require transformation first.^29^, ^33^, ^56^ Similar to the predictor effects, a prediction interval can then be constructed for the performance estimates, which provides an interval of likely values that the performance statistic will have in a new cluster.

Besides allowing one to obtain an average estimate of performance, meta‐analysis is particularly helpful for investigating the presence of heterogeneity of prediction model performance and any possible causes thereof.^10^, ^31^, ^33^ Prediction model performance may vary across clusters due to imprecision or bias of the regression coefficients or performance estimates, or due to the variation in population characteristics. Disentangling these various sources of variation is necessary when inferring on the model's potential generalizability to different settings and populations.

Finally, if the average performance and heterogeneity of the performance of the prediction model are deemed adequate, that is it is considered likely that performance will be adequate in a new cluster, a so called global model may be developed by estimating the coefficients for the predictors on the data of all available clusters. In this final step no clusters are left out, in order to minimize the variability of the estimates of the coefficients.^25^

### 2.3 Motivating example: Diagnosis of deep vein thrombosis

Patients with a deep vein thrombosis (DVT) have an increased risk of post‐thrombotic syndrome and pulmonary embolism, which can be fatal.^57^ In the majority of patients in whom DVT is suspected, no DVT is present on advanced (reference) testing.^58^ For illustrative purposes, we here consider the diagnosis of DVT in patients that are suspected of having DVT and use the IPD of 10 014 patients from 11 studies,^59^ where each study is considered one cluster (Tables 3 and 4). In each cluster separately, we estimated a binary logistic regression model with three prespecified predictors: history of malignancy (yes/no), calf difference (difference in circumference of the calves ≥ 3 cm), recent surgery (yes/no). Preferably, a continuous predictor such as calf difference should not be dichotomized, as this leads to a loss of information. However, the continuous predictor was not available in the data at hand. As some clusters were small, we applied Firth's correction,^60^ which yields unbiased Maximum Likelihood estimates for the coefficients and SEs in small samples^61^ and adjusted the intercept post hoc by re‐estimating it with an unpenalized GLM.^62^ We then applied IECV and adopted a two‐stage approach for prediction model development. The pooled regression coefficients (including the intercept term) from the development clusters were used for generating predictions in the hold‐out cluster. Although Firth's correction still yielded estimates with high variance for the predictor coefficients in some clusters, this was mitigated by performing a meta‐analysis of the regression coefficients.

**TABLE 3:** Clinical characteristics of DVT data

Outcome: DVT		No	Yes	Total
Sex	Female	5174 (83.8)	1001 (16.2)	6175
	Male	2943 (76.7)	896 (23.3)	3839
Malignancy	No	7600 (82.8)	1581 (17.2)	9181
	Yes	517 (62.1)	316 (37.9)	833
Recent surgery	No	7333 (82.4)	1569 (17.6)	8902
	Yes	784 (70.5)	328 (29.5)	1112
Leg trauma	No	5210 (77.1)	1544 (22.9)	6754
	Yes	2907 (89.2)	353 (10.8)	3260
Vein distension	No	7257 (82.5)	1538 (17.5)	8795
	Yes	860 (70.5)	359 (29.5)	1219
Calf difference >3 cm	No	6160 (88.0)	843 (12.0)	7003
	Yes	1957 (65.0)	1054 (35.0)	3011
D‐dimer abnormal	No	4392 (97.0)	137 (3.0)	4529
	Yes	3725 (67.9)	1760 (32.1)	5485
Age	Mean (SD)	58.8 (17.4)	61.1 (17.1)	10 014
Duration of symptoms	Mean (SD)	22.8 (45.5)	27.0 (60.5)	10 014

Abbreviation: DVT, deep vein thrombosis.

**TABLE 4:** Estimated regression coefficients (and SEs) for predicting DVT in each of 11 clusters

Cluster	Intercept	Malignancy	Calf difference	Surgery
1	−2.46 (0.14)	0.90 (0.33)	1.17 (0.19)	0.04 (0.35)
2	−0.95 (0.11)	0.31 (0.24)	0.98 (0.15)	0.17 (0.25)
3	−2.92 (0.44)	1.57 (0.87)	1.59 (0.50)	1.73 (0.54)
4	−1.92 (0.09)	0.63 (0.16)	1.68 (0.13)	0.83 (0.17)
5	−2.27 (0.16)	0.24 (0.42)	1.03 (0.20)	0.52 (0.26)
6	−2.25 (0.12)	1.23 (0.30)	1.40 (0.17)	0.51 (0.21)
7	−3.18 (0.13)	1.69 (0.22)	1.41 (0.19)	0.26 (0.31)
8	−1.72 (0.18)	1.02 (0.58)	1.24 (0.27)	0.78 (0.51)
9	−2.01 (0.11)	0.80 (0.25)	1.25 (0.14)	0.37 (0.19)
10	−2.16 (0.18)	1.04 (0.46)	0.65 (0.34)	0.79 (0.35)
11	−2.30 (0.19)	1.65 (0.26)	1.32 (0.23)	0.82 (0.27)
Summary estimate	−2.17 (0.18)	0.98 (0.17)	1.27 (0.08)	0.55 (0.09)
Approximate 95*%* prediction interval	−3.33:−1.01	0.08:1.88	0.86:1.67	0.20:0.90

*Note*: Malignancy: history of malignancy; Calf difference: difference in circumference of calves ≥ 3 cm; Surgery: recent surgery. Summary estimates and prediction intervals for global model.

Abbreviation: DVT, deep vein thrombosis.

Results in Table 4 reveal that estimates for the predictor effects were very heterogeneous across the included clusters. For example, the coefficient for malignancy was 0.90 (SE: 0.33) in cluster 1 and 1.69 (SE: 0.22) in cluster 7. Similarly, the coefficient for calf difference was 0.98 (SE: 0.15) in cluster 2 and 1.68 (SE: 0.13) in cluster 4. As indicated in Table 5 this also resulted in heterogeneous model performance estimates across hold‐out clusters. Although calibration was good on average, it was highly variable in individual clusters. For instance, whereas the summary calibration‐in‐the‐large equaled 0.03 (95% CI: −0.33 to 0.39), meaning that calibration‐in‐the‐large was very good on average, the calibration‐in‐the‐large's approximate 95% prediction interval (PI) ranged from −1.22 to 1.27, thereby indicating the presence of heterogeneity of the prediction model's calibration. Similarly, the calibration of the linear predictors was very good on average, as the calibration slope (also estimated with Firth's correction) equaled 1.00 (95% CI: 0.83 to 1.16), whereas the approximate 95% PI for the calibration slope ranged from 0.53 to 1.46. Further, the c‐statistic equaled 0.68 (95% CI: 0.65 to 0.71) and was also substantially heterogeneous across clusters (approximate 95% PI: 0.60 to 0.75).

**TABLE 5:** Internal‐external cross‐validation performance estimates and SEs for the predefined model for predicting DVT

Hold‐out cluster for validation	Slope (SE)	CIL (SE)	c‐Statistic (SE)
1	0.86 (0.15)	−0.44 (0.10)	0.65 (0.02)
2	0.63 (0.11)	1.06 (0.08)	0.63 (0.02)
3	1.49 (0.35)	−0.24 (0.22)	0.78 (0.05)
4	1.18 (0.10)	0.43 (0.06)	0.72 (0.01)
5	0.73 (0.15)	−0.33 (0.10)	0.65 (0.02)
6	1.12 (0.14)	−0.00 (0.08)	0.70 (0.02)
7	1.24 (0.14)	−0.93 (0.09)	0.71 (0.02)
8	1.02 (0.24)	0.51 (0.13)	0.67 (0.03)
9	0.92 (0.11)	0.12 (0.07)	0.68 (0.02)
10	0.71 (0.27)	−0.09 (0.14)	0.62 (0.04)
11	1.27 (0.17)	0.15 (0.11)	0.74 (0.03)
Summary estimates	1.00 (0.08)	0.03 (0.16)	0.68 (0.06)
Approximate 95*%* prediction interval	0.53:1.46	−1.22:1.27	0.60:0.75

*Note*: Slope: calibration slope.

Abbreviations: CIL, calibration‐in‐the‐large; DVT, deep vein thrombosis.

On overall, the IECV showed that the modeling strategy was unlikely to yield a prediction model with good generalizability. Substantial revision would be necessary to improve the model's average discrimination performance and to reduce the heterogeneity of its calibration and discrimination performance. A possible approach would be to refine the original modeling strategy by altering the set of included predictors and by considering interaction effects and/or nonlinear terms. It is generally recommended to define a set of predictors and functions and interactions thereof on beforehand. Recommendations for this are given elsewhere.^2^, ^63^, ^64^, ^65^

Subsequently, the revised model should be validated again, after which other revisions may be decided and so forth. It may be clear that this strategy is very time consuming and may lead to arbitrary choices in predictor selection. For these reasons we propose a formal framework for predictor selection in the context of heterogeneity of performance across clusters in the next section. We address methods that aim to reduce heterogeneity of performance, improve the average performance and a combination thereof. The code used to apply our methodology as presented in this article is available on Github (https://github.com/VMTdeJong/SIECV‐DVT).

## 3 STEPWISE INTERNAL‐EXTERNAL CROSS‐VALIDATION FOR MODEL DEVELOPMENT

In the previous section, we described the purpose of IECV to assess the generalizability of a prediction model that is generated by a predefined modeling strategy. Here, we propose to extend IECV to optimize model generalizability *during its development*. We here describe the algorithm for the situation that IECV will be used to expand an empty (intercept only) model by iteratively adding predictors, functions of predictors and interaction effects.

We assume that these candidate predictors (and functions thereof) have already been selected through clinical reasoning and evidence from previous studies, such as reviews of prediction models and prognostic factor studies. The proposed methodology is not intended for dimensionality reduction (eg, variable selection in settings with large P, small N), but is used to assess the heterogeneity of the predictive performance resulting from including these in the prediction model.

The approach readily generalizes to the expansion or reduction of a given model. In this stepwise IECV (SIECV) for prediction model development models are estimated, validated in external data sets, and updated in an iterative process, as follows.

Denote the data from the *k* ^th^ cluster by *S*_*k*_, and the data from a set of clusters excluding cluster *h* by *S*_(*h*)_. Let *p*, *p* = 0, 1, … , *P* be indicators to denote the candidate predictors (or functions thereof), where *p* = 0 indicates none. The algorithm consists of up to *I* model adaptation cycles, where *I* generally equals *P*, the number of predictors available for inclusion in the model. Then, let *P*_*r*_(*i*) denote the set of candidate predictors for inclusion, where *P*_*r*_(1) = {1, 2, … , *P*} and *P*_*r*_(0) = {0}.

Further, let *M*_*i*, *p*_ denote the models in cycle *i* with added predictor *p* in the stepwise process. Let *M*_*i*, *p*, (*h*)_ denote a model estimated on data from all clusters excluding *S*_*h*_. Let ${\hat{Z}}_{i,p,h}$ be an estimate of performance (ie, a loss function) of model *M*_*i*, *p*, (*h*)_ in cluster h, such as the mean squared error. Let ${Â}_{i,p}$ be the estimate of a loss function (ie, an aggregated loss function or an estimate of heterogeneity, further described in Section 3.2) in cycle *i* for a model extended with predictor *p*, and let *c* indicate a predictor *p* that has minimal ${Â}_{i,p}$, such that *M*_*i*, *c*_ is the model with best generalizability in cycle *i*. Then, the algorithm is defined as follows and starts at cycle *i* = 0:

1. For all *p* in *P*_*r*_(*i*):
  (a) Extend model *M*_*i* − 1, *c*_ with predictor *p* to generate new model *M*_*i*, *p*_.
  (b) For *h*, *h* = 1, … , *K*:
    (i) Estimate the model *M*_*i*, *p*, (*h*)_ on *S*_(*h*)_, preferably while taking clustering within clusters into account.
    (ii) Predict ${ŷ}_{i,p,h,j}$ for individual participants in hold‐out sample *S*_*k*_.
    (iii) Estimate performance measure ${\hat{Z}}_{i,p,h}$ and its SE $\hat{\text{SE}}({\hat{Z}}_{i,p,h})$ for predictions ${ŷ}_{i,p,h,j}$ in *S*_*h*_.
  (c) Estimate aggregated loss function ${Â}_{i,p}$ on ${\hat{Z}}_{i,p,1},\ldots ,{\hat{Z}}_{i,p,K}$ and $\hat{\text{SE}}({\hat{Z}}_{i,p,1}),\ldots ,\hat{\text{SE}}({\hat{Z}}_{i,p,K})$.
2. Find the minimal ${Â}_{i,p}$ in this cycle. Denote this by ${Â}_{i,c}$ and its corresponding model by *M*_*i*, *c*_.
3. The first condition that is satisfied:
  (a) If *i* = 0, continue to step 1.
  (b) Else, if ${Â}_{i,c}\geq {Â}_{i-1,c}$, the algorithm stops and *M*_*i* − 1, *c*_ is returned as the final model.
  (c) Else, if *i* = *I*, the algorithm stops and *M*_*i*, *c*_ is returned as the final model.
  (d) Else, remove predictor *c* from the candidate predictor set *P*_*r*_(*i*), increment *i* by 1 and continue to step 1.

Finally, if the performance of model *M*_*i*, *c*_ is deemed satisfactory, a so called global model is generated by estimating the coefficients for the predictors in *M*_*i*, *c*_ on all available data. No clusters are left out in this final cycle, to reduce the variance of the estimates of the coefficients.^25^

This global model however, is at risk of overfitting as a result of small sample bias, unless the sample is sufficiently large and the event rate sufficiently high, even if no selection of predictors were applied.^35^, ^37^, ^66^ To account for this, the prediction model could be fitted with penalized regression, such as Firth's regression. To reduce the variance of the estimated regression coefficients, the ridge penalty could be applied instead, or one could opt for a fully Bayesian approach.

By considering the candidate predictors for inclusion, however, the prediction model is at further risk of overfitting.^2^, ^64^ A straightforward adjustment for overfitting could be achieved with the calibration slope and calibration‐in‐the‐large.^67^ In step 1 (b) iii of the final cycle these could be estimated and then summary meta‐analyses estimates could be computed. The final model coefficients (excluding the intercept) would then be multiplied by the summary calibration slope, whereas the summary calibration‐in‐the‐large would subsequently be estimated and added to the global model's intercept, thereby yielding a final model. Ideally, however, the entire model selection procedure is to be performed within an additional bootstrap or cross‐validation procedure,^68^ as this would account for any overfitting introduced by the SIECV itself. Alternatively, heuristic shrinkage, that shrinks the coefficients by a function of the number of predictors considered, may be applied.^2^, ^64^, ^69^

### 3.1 Extensions

Throughout this article, we work from the perspective that an entirely new prediction model is to be developed. However, our proposed framework readily encompasses model redevelopment including the adding and removal of predictor terms. Predictors that are known from previous research to have a strong predictive effect can then be forced in the model instead of applying a selection strategy to these predictors, as is generally recommended.^65^ Selection of predictor effects may then also be performed with a backward procedure starting with all candidate predictors and their transformations or interactions, rather than forward. Though, this may yield issues in the estimation when many predictor effects are considered, especially when random effects are applied. In a simulation study (described in online supporting information A, code on Github [https://github.com/VMTdeJong/SIECV‐sim‐DVT]), the forward and backward SIECV methods had similar out‐of‐sample performance, both in terms of average performance and generalizability thereof.

Further, similar to IECV for model validation we may adopt a one‐ or two‐stage approach (Section 2.1) for model estimation. To enhance the interpretability of a prediction model, it is generally recommended to include main effects for variables that are included as interaction effects. Hence, the algorithm may be adjusted so that this is always the case by forcing the main effect to remain in the model whenever an interaction with another variable is included.

### 3.2 Quantifying model generalizability

The SIECV algorithm requires specification of an aggregated loss function (*A*_*i*, *p*_) that is to be minimized, in order to optimize generalizability of performance across clusters. Here, we consider parametric and nonparametric aggregated loss functions, that vary with respect to the importance they place on the average and heterogeneity of performance.

#### 3.2.1 Ignoring heterogeneity

As a first step, we consider a naive estimator of predictive performance across hold‐out data sets from different clusters, that ignores variation within and across clusters. This approach may be reasonable when the clusters are very large and of similar size, and when the effect of clustering is negligible (that is when the intraclass correlation coefficient is near zero). The overall performance is then given by the mean performance across clusters. For instance, when optimizing the mean square error (MSE, or Brier score for categorical outcomes), we can apply the following aggregated loss function:

(4) $$\hat{A_{i,p}^{M}}=\frac{1}{K}\overset{K}{\underset{h=1}{\sum }}{\hat{Z}}_{i,p,h}.$$

#### 3.2.2 Weighted meta‐analysis

To incorporate the uncertainty of the predictive performance estimates into an aggregated loss function, it may be more appropriate to adopt a weighting procedure. The meta‐analysis framework (see Section 2.1) therefore appears an appealing choice. A straightforward extension to Equation (4) would be to apply the weighting procedure in described in Equations (2) and (3). This allows to minimize the prediction error in an “average” cluster, but still does not attempt to optimize their stability across clusters. As a result, it is possible that developed models perform well on average, but require substantial local revisions before implementation. To reduce the need for local revisions, the aggregated loss function should account not only for the average performance, but also for its variation across clusters. For this reason, we propose an extension that combines both sources of error:

(5) $$\hat{A_{i,p}^{{\text{RE}}_{\lambda }}}=\lambda {\hat{Z}}_{i,p}^{\text{RE}}+(1-\lambda ){\hat{\tau }}_{i,p},$$

where λ is a hyperparameter that defines the impact of random effects meta‐analysis summary estimate of performance ${\hat{Z}}_{i,p}^{\text{RE}}$ and heterogeneity estimate ${\hat{\tau }}_{i,p}$ on aggregated loss function $\hat{A_{i,p}^{{\text{RE}}_{\lambda }}}$. This is a parameter that is to be chosen on beforehand, where its value should depend on the relative importance of average and heterogeneity of performance. In the simplest case we let λ = 1, such that the estimate for generalizability is given by the mean of the distribution of performance, $\hat{A_{i,p}^{{\text{RE}}_{1}}}={\hat{Z}}_{i,p}^{\text{RE}}$. Alternatively, if desired, we can set λ to 0, such that we can inform the selection of predictors solely based on the reduction in heterogeneity of performance, yielding $\hat{A_{i,p}^{{\text{RE}}_{0}}}={\hat{\tau }}_{i,p}$. Finally, we consider the case where heterogeneity of performance and average performance are given equal weighting by setting $\lambda =\frac{1}{2}$, such that $\hat{A_{i,p}^{{\text{RE}}_{1/2}}}=\frac{1}{2}{\hat{Z}}_{i,p}^{\text{RE}}+\frac{1}{2}{\hat{\tau }}_{i,p}$.

This equation can be seen as an extension of the bias‐variance decomposition of the MSE where we now have a summation of squared bias, within‐cluster variance and between‐cluster variance. If p are considered estimators for y, then the MSE for p can be shown to be: $\text{MSE}(p)=\mathrm{var}(p)+\mathrm{Bias}{\left(p,y\right)}^{2}$. As ${\hat{\tau }}^{2}$ is the estimate of the between cluster variance of MSE(p), that is, var_*bs*_, the estimator $\hat{A_{i,p}^{{\text{RE}}_{1/2}}}$ estimator can be interpreted as the mean of:

(6) $$\frac{1}{2}\lambda (\mathrm{var}(p)+\text{Bias}{\left(p,y\right)}^{2})+\frac{1}{2}(1-\lambda )({\mathrm{var}}_{bc}(\mathrm{var}(p)+\text{Bias}{\left(p,y\right)}^{2})).$$

Similar to the meta‐analysis of prediction model coefficients discussed in Section 2.1 it is important to consider the estimation of between‐cluster heterogeneity in prediction model performance. For this reason, we recommend a random effects meta‐analysis to summarize estimates of prediction model performance. As discussed in Section 2.1, the random effects meta‐analysis model is preferably estimated with REML or the Paule‐Mandel method. Estimates of between‐cluster heterogeneity can then be used to derive an approximate prediction interval that provides information on the likely values that model performance may take in a new cluster. A sufficient number of clusters each with sufficient observations as well as an adequate estimation method are necessary to make this approximate prediction interval informative

#### 3.2.3 Variability of performance across data sets

In the meta‐analysis approach, the evidence from small clusters is downweighted to attain an estimate of the mean of the distribution of performance. Yet this distribution might not be of central importance. Instead, all clusters might be considered of equal importance. Then we may instead apply a measure of variability directly to the performance estimates, for instance the SD $\hat{A_{i,p}^{\text{SD}}}=SD({\hat{Z}}_{i,p,1},\ldots ,{\hat{Z}}_{i,p,K})$.

Alternatively, if no assumptions can be made on the distribution of the predictive performance statistics, we may apply a nonparametric measure. For example, when using Gini's Mean Difference we have:^70^, ^71^

(7) $${\hat{A^{\text{Gini}}}}_{i,p}=\frac{2}{K(K-1)}\underset{1\leq h\leq v\leq K}{\sum }|{\hat{Z}}_{i,p,v}-{\hat{Z}}_{i,p,h}|.$$

## 4 MOTIVATING EXAMPLE 2: UPDATING A MODEL FOR DIAGNOSING DVT

The prediction model developed in Section 2.3 had a rather heterogeneous performance across validation clusters and was lacking in average discrimination performance. This heterogeneity of performance implies that although the outcome may be predicted well in individuals in some clusters, which may be helpful in diagnosis, it may be unsatisfactory for individuals in other clusters. The prediction model performance heterogeneity across the 11 clusters may be explained by differences in (measured and unmeasured) predictor distributions and true predictor effects. Therefore we here consider whether additional predictors and interaction effects might explain such differences. Whereas individual clusters may lack the sample size to detect nonlinear effects or may lead to highly variable predictor effects, this is more feasible in pooled studies such as for an IPD‐MA and in large healthcare data bases.

Briefly, we considered the following ten additional candidate predictors to extend the model from Section 2.3: sex, absence of leg trauma, absence of leg trauma × recent surgery (ie, an interaction effect), vein distension, log of duration of symptoms, age/25 (ie, divided by 25, to increase the absolute value of its coefficient), age/25 squared, age/25 × malignancy, abnormal d‐dimer value and abnormal d‐dimer × sex. As we developed this model for illustrative purposes only, we applied multiple imputation for missing data using a joint model with random effects, but have used only one imputed data set for analyses.^30^, ^72^ An overview of the missing data per variable per cluster is given in online supporting information B.

We recommend that the inclusion of each candidate predictor (or transformation thereof) be carefully considered with respect to the improvements in generalizability of the model performance on the one hand, and the cost and harms relating to the measurement of the predictor on the other. Further, it is generally recommended that predictors that are known from previous research to have a strong predictive effect should be forced in the model instead of applying a selection strategy to these predictors.^65^ Accordingly, we force malignancy, calf difference and surgery as predictors into the model in this example. We apply our methodology to illustrate how each of the strategies regarding heterogeneity of performance leads to different model specifications, and thereby to differing average and heterogeneity of performance. To assess the generalizability of prediction models that use these predictor functions, we follow the SIECV strategy for model development that we developed in Section 3, apply the MSE (ie, Brier score) to the predicted probabilities in the hold‐out clusters and apply the aggregated loss functions (measures of heterogeneity of performance) on the MSE estimates and SEs thereof, to select predictors as outlined in Section 3.2.

The six applied aggregated loss functions lead to models with four different predictor function specifications (Table 6), as the strategy that ignored clustering (*A* ^M^) when estimating generalizability of performance lead to the same model specification as the strategy that optimized the meta‐analytic mean of performance ($A^{{\text{RE}}_{1}}$), and the meta‐analysis strategy that placed equal importance on heterogeneity of performance and average performance ($A^{{\text{RE}}_{1/2}}$) lead to the same model specification as the *A* ^SD^ strategy. As the SIECV allows for the estimation of any performance statistic, we assessed discriminatory performance with the c‐statistic, and calibration with the calibration slope and calibration‐in‐the‐large, for the final model for each aggregated loss function. Subsequently, we summarized the performance and heterogeneity thereof with univariate random effects meta‐analyses.

**TABLE 6:** Estimated regression coefficients of seven models for predicting DVT estimated with (S)IECV

Predictor	None	*A* ^M^	$A^{{\text{RE}}_{1}}$	$A^{{\text{RE}}_{1/2}}$	$A^{{\text{RE}}_{0}}$	*A* ^SD^	*A* ^Gini^
Intercept	−2.17	−3.54	−3.54	−5.13	−5.00	−5.13	−3.99
Malignancy	0.98	0.76	0.76	1.64	1.68	1.64	2.05
Calf difference	1.26	1.13	1.13	1.38	1.34	1.38	1.07
Surgery	0.55	−0.04	−0.04	0.25	0.25	0.25	0.34
D‐dimer positive		2.76	2.76	2.99	2.94	2.99	2.81
Age/25		−0.22	−0.22				
Vein distension		0.46	0.46				
Surgery × no leg trauma		0.68	0.68				
No leg trauma				0.95	0.96	0.95	
(Age/25)^2^				−0.02		−0.02	
Male				0.32	0.36	0.32	0.52
D‐dimer positive × male				−0.20	−0.24	−0.2	−0.28
Malignancy × age/25				−0.32	−0.35	−0.32	−0.50

*Note*: Malignancy: history of malignancy; Calf difference: difference in circumference of calves ≥ 3 cm; Surgery: recent surgery; Age/25: age divided by 25; Duration: duration of symptoms; None: model with no predictor selection. Empty cells indicate the predictor was not selected for inclusion in the corresponding model. The predictor functions that were not selected in any final model are omitted from this table. Summary predictor effects were estimated by the Dersimonian and Laird method, as REML did not converge for the estimation of some models. Although REML has better theoretical properties for the heterogeneity estimate, the difference for the summary effects (presented here) is limited.

Abbreviations: *A* ^Gini^, Gini's mean difference of MSE; *A* ^M^, average of MSE; $A^{{\text{RE}}_{1}}$, random effects meta‐analytic estimate of mean of MSE; $A^{{\text{RE}}_{0}}$, random effects meta‐analytic estimate of heterogeneity of distribution of MSE; $A^{{\text{RE}}_{1/2}}$, sum of random effects meta‐analytic estimates of mean and heterogeneity of distribution of MSE; *A* ^SD^, standard deviation of MSE; DVT, deep vein thrombosis; MSE, mean square error; (S)IECV, (stepwise) internal‐external cross‐validation.

**FIGURE 1:**
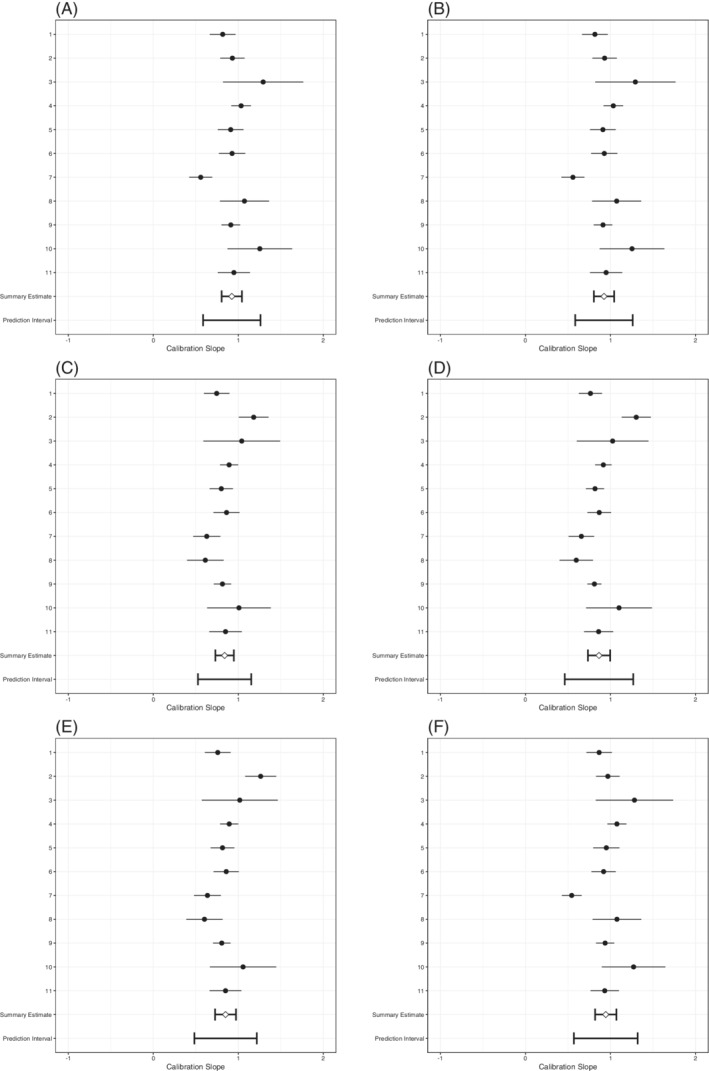
Forest plots of SIECV estimates of calibration slopes of six strategies for predicting DVT

In terms of calibration slopes (also estimated with Firth's correction), all strategies showed some overfit (summary calibration slope < 1), though to varying degrees (Table 7, Figure 1). Slopes <1 imply that the estimated slopes were too large (the log odds ratios deviated too far from 0), which yielded predictions for individuals that were too extreme. The linear predictors in the *A* ^SD^ strategy and the meta‐analytic strategy that combined heterogeneity of performance and average performance ($A^{{\text{RE}}_{1/2}}$) were the worst calibrated (calibration slope of 0.85), and the *A* ^Gini^ strategy the best (0.94).

**TABLE 7:** Meta‐analysis summary estimates of SIECV performance of six strategies for predicting DVT

Measure	Strategy	$A^{{\text{RE}}_{1}}$	95*%* CI	95*%* PI
Calibration slope	None	1.00	0.83:1.16	0.53:1.46
	*A* ^M^	0.92	0.80:1.04	0.59:1.26
	$A^{{\text{RE}}_{1}}$	0.92	0.80:1.04	0.59:1.26
	$A^{{\text{RE}}_{1/2}}$	0.85	0.73:0.97	0.48:1.22
	$A^{{\text{RE}}_{0}}$	0.87	0.73:1.00	0.46:1.27
	*A* ^SD^	0.85	0.73:0.97	0.48;1.22
	*A* ^Gini^	0.94	0.82:1.07	0.57:1.32
Calibration‐in‐the‐large	None	0.03	−0.33:0.39	−1.22:1.27
	*A* ^M^	−0.06	−0.46:0.35	−1.47:1.35
	$A^{{\text{RE}}_{1}}$	−0.06	−0.46:0.35	−1.47:1.35
	$A^{{\text{RE}}_{1/2}}$	0.16	−0.25:0.58	−1.27:1.60
	$A^{{\text{RE}}_{0}}$	−0.04	−0.47:0.39	−1.51:1.43
	*A* ^SD^	0.16	−0.25:0.58	−1.27:1.60
	*A* ^Gini^	−0.20	−0.61:0.21	−1.63:1.23
c‐Statistic	None	0.68	0.65:0.71	0.60:0.75
	*A* ^M^	0.81	0.78:0.84	0.70:0.89
	$A^{{\text{RE}}_{1}}$	0.81	0.78:0.84	0.70:0.89
	$A^{{\text{RE}}_{1/2}}$	0.81	0.79:0.84	0.73:0.88
	$A^{{\text{RE}}_{0}}$	0.81	0.79:0.84	0.71:0.89
	*A* ^SD^	0.81	0.79:0.84	0.73:0.88
	*A* ^Gini^	0.81	0.77:0.84	0.68:0.90

*Note*: None: model with no predictor selection.

Abbreviations: 95*%* CI, 95% confidence interval; 95*%* PI, the random effects meta‐analysis approximate 95% prediction intervals lower and upper bound; *A* ^Gini^, Gini's mean difference of MSE; *A* ^M^, average of MSE; $A^{{\text{RE}}_{1}}$, random effects meta‐analytic estimate of mean of MSE; $A^{{\text{RE}}_{0}}$, random effects meta‐analytic estimate of heterogeneity of distribution of MSE; $A^{{\text{RE}}_{1/2}}$, sum of random effects meta‐analytic estimates of mean and heterogeneity of distribution of MSE; *A* ^SD^, standard deviation of MSE; DVT, deep vein thrombosis; MSE, mean square error; SIECV, stepwise internal‐external cross‐validation.

There was substantial heterogeneity in the estimated calibration slopes, especially for the predefined model with no predictor selection. For all strategies, the prediction interval for the calibration slope also included values >1, which implies that for some (future) clusters the log odds ratios will probably not deviate from 0 enough and that predictions for individuals will probably be not extreme enough. The heterogeneity of the calibration slope decreased for all strategies, as compared to the predefined model with no added predictors. This means that for the resulting models there was a decreased need for extensive local updating.

**FIGURE 2:**
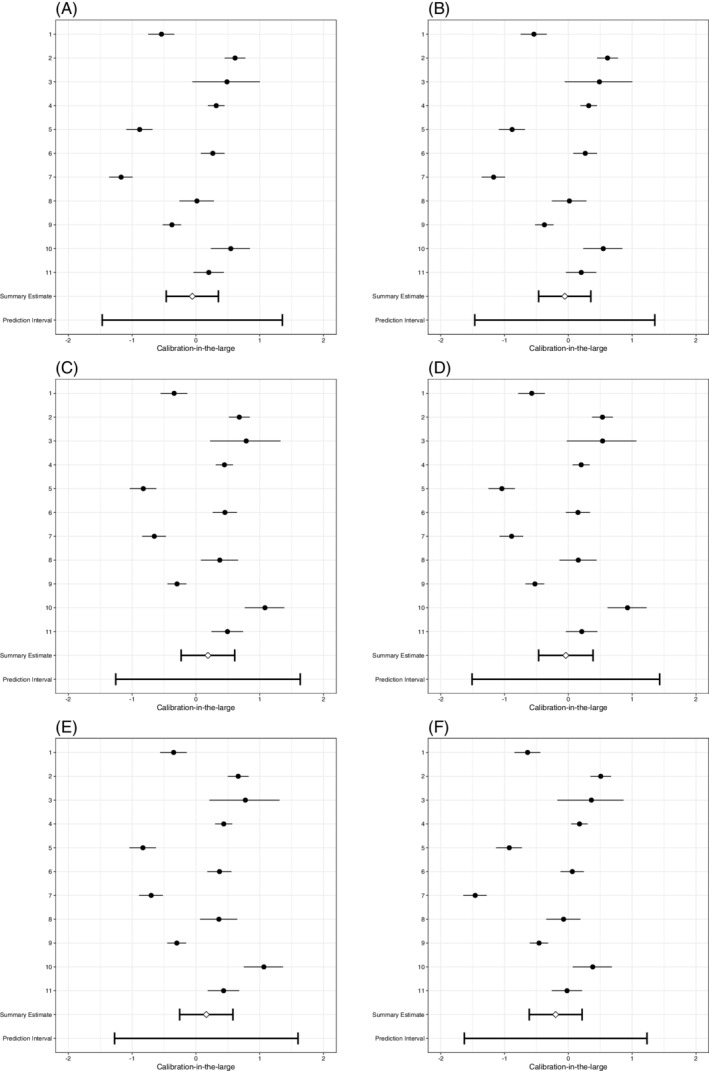
Forest plots of SIECV estimates of calibration‐in‐the‐large of six strategies for predicting DVT

In terms of average calibration‐in‐the‐large, all strategies achieved a reasonable calibration‐in‐the‐large, that is close to zero (Table 7, Figure 2). This means that on average the incidence was predicted accurately. On the other hand, the meta‐analysis of the calibration‐in‐the‐large showed that the heterogeneity of calibration‐in‐the‐large had increased for all modeling strategies, as compared to the predefined model with no added predictors. Hence, a trade‐off occurred between (heterogeneity of) calibration slopes and calibration‐in‐the‐large, and local updating of the intercept will remain necessary.

**FIGURE 3:**
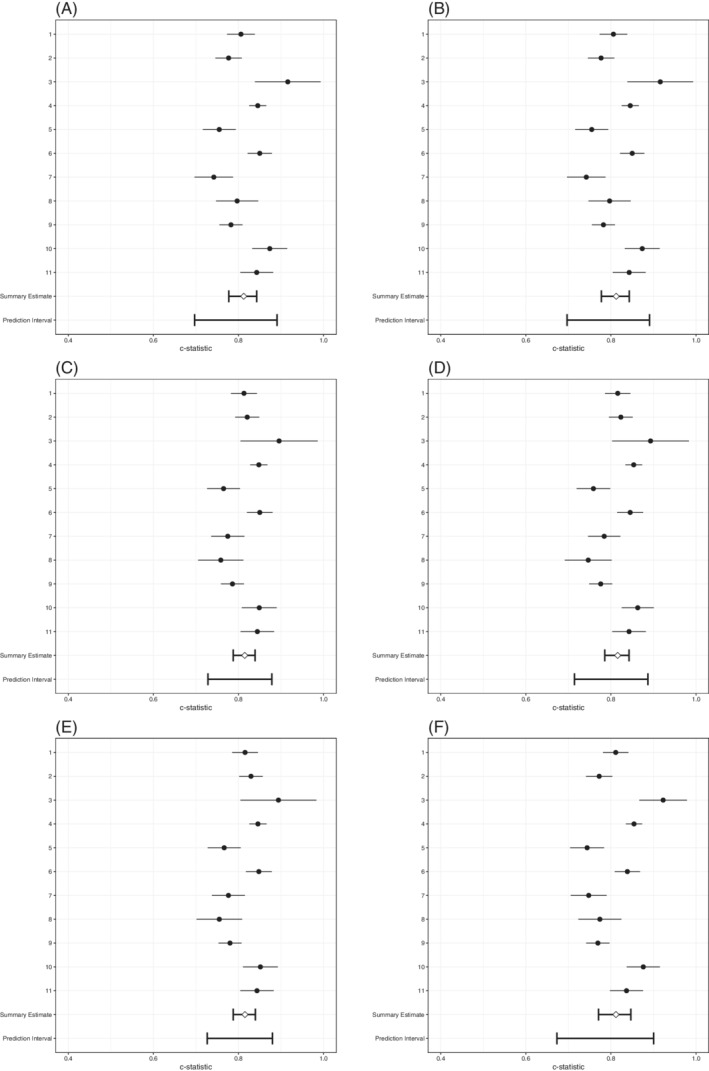
Forest plots of SIECV estimates of c‐statistics of six strategies for predicting DVT

All strategies that applied SIECV achieved an internally‐externally validated c‐statistic value of 0.81 (Table 7, Figure 3). There were small differences in heterogeneity of discrimination performance. The *A* ^SD^ strategy and the strategies that included the meta‐analytic estimate of heterogeneity had smaller values for the heterogeneity of the internally‐externally validated c‐statistic, than the strategies that focused on the mean performance alone (*A* ^M^ and $A^{{\text{RE}}_{1}}$). Further, the *A* ^Gini^ strategy, a non‐meta‐analytic strategy that focuses on heterogeneity of performance, yielded larger heterogeneity of discrimination performance.

As a final step, one must choose which modeling strategy is most likely to yield adequate performance when applied to individuals in a new cluster, if any. Although heterogeneity in the slopes had decreased substantially for all strategies, the prediction intervals still indicated that updating may be necessary. Further, the models resulting from all strategies are likely to need an intercept update. In terms of calibration, it may therefore not be advisable to develop a global model, that is a model developed on all available clusters (without leaving any out). Finally, although the discrimination for all models improved substantially, the diagnostic utility would have to be put into a clinical perspective.

## 5 MOTIVATING EXAMPLE 3: PREDICTING ATRIAL FIBRILLATION

Patients with atrial fibrillation (AF) are at an increased risk for stroke.^73^ Although stroke is usually not fatal, it often results in neurological deficiencies.^74^ In patients with AF the incidence of stroke as well as the incidence of death from stroke can be greatly reduced by oral anticoagulation.^75^

For illustrative purposes, we here consider the development and validation of a binary logistic prediction model to estimate the probability that atrial fibrillation is present in an individual patient. Previously, Audigier et al prepared a simulated data set to mimic the patients from 28 cohorts (clusters, from hereon) of the GREAT consortium.^76^, ^77^ This data set comprises a total of 11 685 patients of which 3335 have AF.

Because some of the clusters are very small and may thereby cause estimation issues during model development or validation, we removed a total of eight clusters in which fewer than 50 patients had the outcome or did not have the outcome. Missing values were imputed using a joint model with random effects.^30^, ^78^ As we apply the methodology for illustrative purposes only, we have used only the first imputed data set for analyses. We subsequently modeled the probability of the presence of atrial fibrillation in 10 873 patients from the remaining 20 clusters (Table 8). To further prevent overfitting, we applied Firth's correction,^60^ and re‐estimated the intercepts with unpenalized maximum likelihood.^62^

**TABLE 8:** Clinical characteristics of AF data

Outcome: AF		No	Yes	Total
Gender	0	4583 (71.3)	1844 (28.7)	6427
	1	3059 (68.8)	1387 (31.2)	4446
BMI	Mean (SD)	27.3 (5.7)	27.4 (5.8)	27.4 (5.7)
Age	Mean (SD)	67.7 (14.1)	73.1 (13.1)	69.3 (14.0)
SBP	Mean (SD)	135.6 (32.2)	135.3 (32.1)	135.5 (32.2)
DBP	Mean (SD)	78.6 (18.3)	79.1 (18.4)	78.7 (18.3)
HR	Mean (SD)	88.0 (25.0)	96.4 (29.0)	90.5 (26.5)
BNP	Mean (SD)	3.0 (0.9)	2.9 (1.0)	2.9 (0.9)

Abbreviations: AF, atrial fibrillation; BMI, body mass index; BNP, brain natriuretic peptide; DBP, diastolic blood pressure; HR, heart rate; SBP, systolic blood pressure; SIECV, stepwise internal‐external cross‐validation.

We considered seven candidate predictors, consisting of gender (binary) and six continuous predictors: body mass index (BMI), age, systolic blood pressure (SBP), diastolic blood pressure (DBP), heart rate (HR), and brain natriuretic peptide (BNP). BMI, age, and HR were divided by 25, and SBP and DBP by 100 to increase the absolute values of their coefficients. For each of the continuous predictors, we considered linear and quadratic terms and applied centering (within clusters) before application of the quadratic function. This was necessary to ensure that the coefficients are stabilized and positive coefficients for quadratic terms represent an increased probability of presence of AF for values that deviate from the mean value. Again, we stress that in practice the in‐ or exclusion of predictor functions should not be based on a statistical criterion alone, but should be carefully considered as discussed in Sections 4 and 6.1.

Here, we implement the proposed predictor selection procedures to illustrate their impact on average performance as well as on generalizability across the different clusters. We follow the SIECV strategy for model development as described in Section 3, apply the MSE to the predicted probabilities in the hold‐out clusters and apply the aggregated loss functions on the MSE estimates and SEs thereof, to select predictors functions as we outlined in Section 3.2.

The six applied aggregated loss functions lead to four different model specifications (Table 9), as the strategy that ignores clustering when quantifying generalizability (*A* ^M^) and the strategy that optimized the meta‐analytic mean of performance ($A^{{\text{RE}}_{1}}$) lead to the same model specification. Further, both meta‐analytic strategies that directly quantified heterogeneity of performance (*A* ^SD^ and *A* ^Gini^) lead to the same model. Again, we assessed performance with the calibration slope, calibration‐in‐the‐large, and c‐statistic, and summarized these and the heterogeneity thereof with univariate random effects meta‐analyses.

**TABLE 9:** Estimated regression coefficients of seven models for predicting AF estimated with SIECV

Predictor	*A* ^M^	$A^{{\text{RE}}_{1}}$	$A^{{\text{RE}}_{1/2}}$	$A^{{\text{RE}}_{0}}$	*A* ^SD^	*A* ^Gini^
Intercept	−0.87	−0.87	−0.85	−0.84	−0.81	−0.81
Gender					−0.08	−0.08
Age/25	0.75	0.75	0.74	0.59	0.62	0.62
HR/25	0.29	0.29	0.32			
SBP/100	−0.59	−0.59				
DBP/100			−0.59			
(Age/25)^2^	−0.17	−0.17	−0.14			
(BMI/25)^2^	0.37	0.37	0.40	0.32	0.32	0.32
(SBP/100)^2^	0.28	0.28				
(BNP)^2^				0.03	0.03	0.03

*Note*: Empty cells indicate the predictor was not selected for inclusion in the corresponding model. The predictor functions that were not selected in any final model are omitted from this table. Summary predictor effects were estimated by the Dersimonian and Laird method, as REML did not converge for the estimation of some models. Although REML has better theoretical properties for the heterogeneity estimate, the difference for the summary effects (presented here) is limited.

Abbreviations: *A* ^Gini^, Gini's mean difference of MSE; *A* ^M^, average of MSE; $A^{{\text{RE}}_{1}}$, random effects meta‐analytic estimate of mean of MSE; $A^{{\text{RE}}_{0}}$, random effects meta‐analytic estimate of heterogeneity of distribution of MSE; $A^{{\text{RE}}_{1/2}}$, sum of random effects meta‐analytic estimates of mean and heterogeneity of distribution of MSE; *A* ^SD^, standard deviation of MSE; AF, atrial fibrillation; BMI, body mass index; BNP, brain natriuretic peptide; DBP, diastolic blood pressure; HR, heart rate; MSE, mean square error; SBP, systolic blood pressure; SIECV, stepwise internal‐external cross‐validation.

**FIGURE 4:**
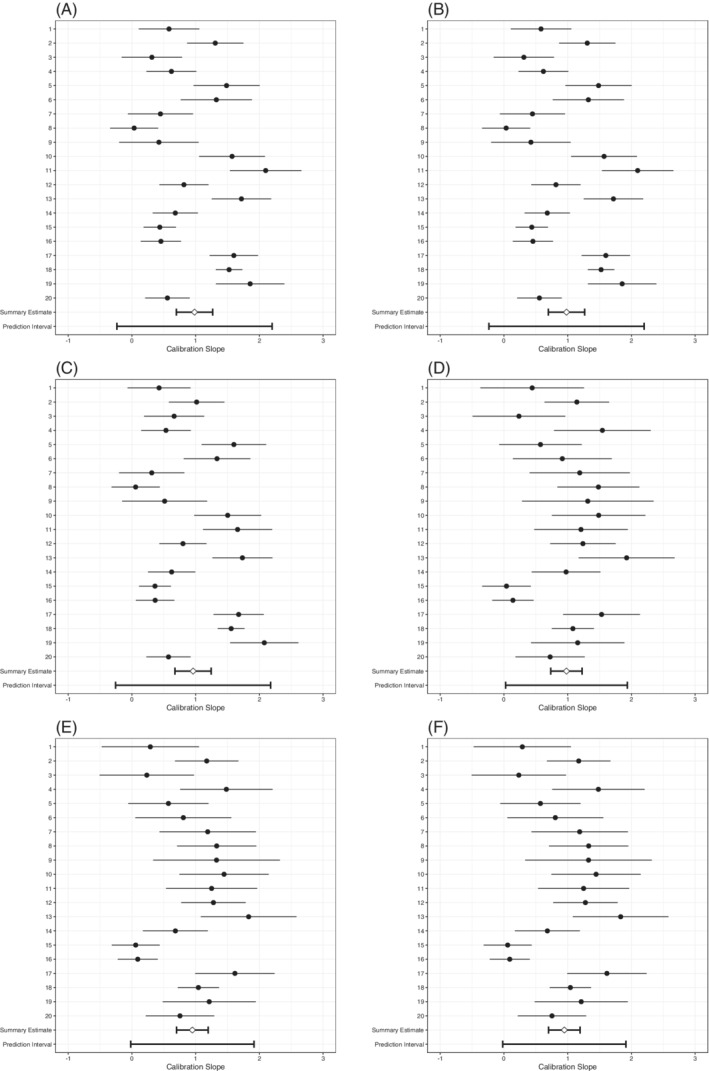
Forest plots of SIECV estimates of calibration slopes of six strategies for predicting AF

In terms of summary calibration slopes (estimated with Firth's correction and then pooled in a meta‐analysis), all strategies were rather well calibrated, showing only minor overfit (Table 10, Figure 4). However, there was substantial heterogeneity of the calibration slopes. The approximate 95% prediction interval of the calibration slopes of the models for the *A* ^M^ and $A^{{\text{RE}}_{1}}$ were the widest, as the upper bound reached 2.20 and the lower bound was estimated at a −0.24, as well as the slopes for $A^{{\text{RE}}_{1/2}}$ which were −0.26 to 2.18. These negative values for the lower bound imply that the predictive effect for the models might be reversed in some clusters: individuals with AF received lower probabilities of AF than individuals without AF in these clusters. This means that for each of these models, there was still a need for extensive updating or model redevelopment.

**TABLE 10:** Meta‐analysis summary estimates of SIECV performance of six strategies for predicting AF

Measure	Strategy	$A^{{\text{RE}}_{1}}$	95*%* CI	95*%* PI
Calibration slope	*A* ^M^	0.98	0.70:1.27	−0.24:2.20
	$A^{{\text{RE}}_{1}}$	0.98	0.70:1.27	−0.24:2.20
	$A^{{\text{RE}}_{1/2}}$	0.96	0.68:1.24	−0.26:2.18
	$A^{{\text{RE}}_{0}}$	0.98	0.74:1.23	0.03:1.94
	*A* ^SD^	0.95	0.70:1.20	−0.02:1.91
	*A* ^Gini^	0.95	0.70:1.20	−0.02:1.91
Calibration intercept	*A* ^M^	0.04	−0.24:0.33	−1.25:1.34
	$A^{{\text{RE}}_{1}}$	0.04	−0.24:0.33	−1.25:1.34
	$A^{{\text{RE}}_{1/2}}$	0.04	−0.25:0.32	−1.25:1.33
	$A^{{\text{RE}}_{0}}$	0.00	−0.28:0.29	−1.28:1.28
	*A* ^SD^	0.00	−0.28:0.29	−1.28:1.28
	*A* ^Gini^	0.00	−0.28:0.29	−1.28:1.28
c‐Statistic	*A* ^M^	0.62	0.58:0.65	0.46:0.75
	$A^{{\text{RE}}_{1}}$	0.62	0.58:0.65	0.46:0.75
	$A^{{\text{RE}}_{1/2}}$	0.62	0.58:0.65	0.46:0.75
	$A^{{\text{RE}}_{0}}$	0.58	0.56:0.60	0.51:0.65
	*A* ^SD^	0.58	0.56:0.60	0.49:0.66
	*A* ^Gini^	0.58	0.56:0.60	0.49:0.66

Abbreviations: 95*%* CI, 95% confidence interval; 95*%* PI, the random effects meta‐analysis approximate 95% prediction intervals lower and upper bound; *A* ^Gini^, Gini's mean difference of MSE; *A* ^M^, average of MSE; $A^{{\text{RE}}_{1}}$, random effects meta‐analytic estimate of mean of MSE; $A^{{\text{RE}}_{0}}$, random effects meta‐analytic estimate of heterogeneity of distribution of MSE; $A^{{\text{RE}}_{1/2}}$, sum of random effects meta‐analytic estimates of mean and heterogeneity of distribution of MSE; *A* ^SD^, standard deviation of MSE; AF, atrial fibrillation; MSE, mean square error; SIECV, stepwise internal‐external cross‐validation.

**FIGURE 5:**
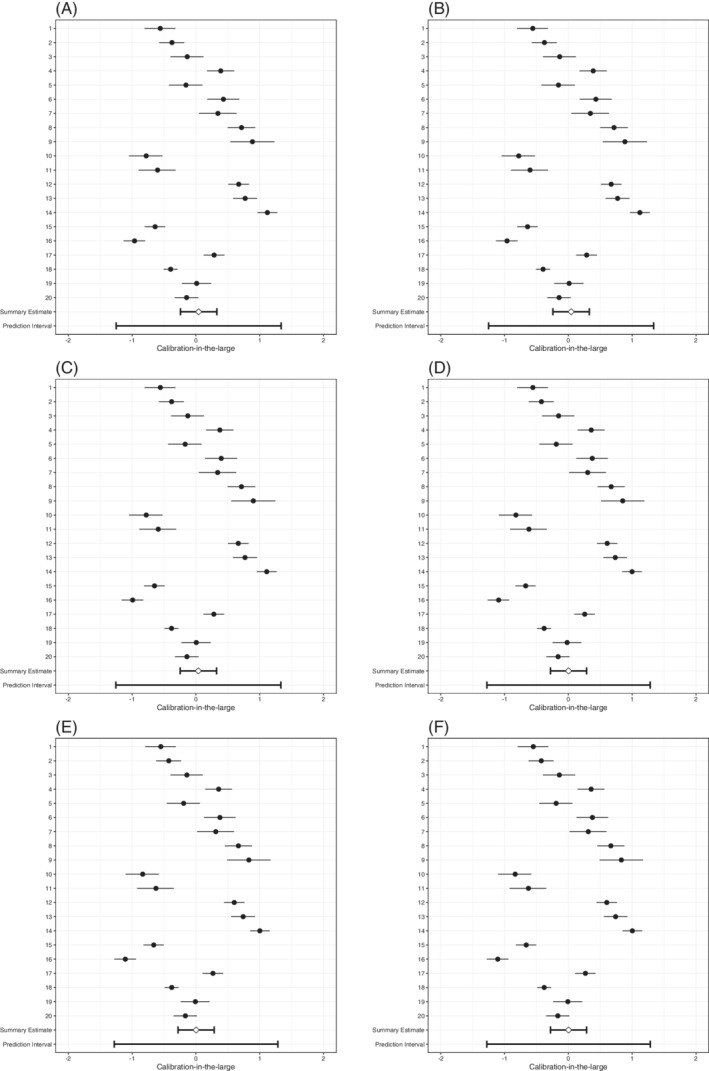
Forest plots of SIECV estimates of calibration‐in‐the‐large of six strategies for predicting AF

Calibration‐in‐the‐large was (near) perfect (Table 10, Figure 5). The $A^{{\text{RE}}_{0}}$, *A* ^SD^, and *A* ^Gini^ strategies all achieved a calibration‐in‐the‐large of 0.00 (95% CI: −0.28 to 0.29), whereas those of *A* ^M^ and $A^{{\text{RE}}_{1}}$ were hardly different with 0.04 (95% CI: −0.24 to 0.33), and neither was the interval for $A^{{\text{RE}}_{1/2}}$ with 0.04 (95% CI: −0.25 to 0.32). Again, there was large heterogeneity in calibration‐in‐the‐large, as shown by the approximate 95% prediction intervals of the calibration‐in‐the‐large. This means that for each of these models there was still a need for intercept updating they may be used.

**FIGURE 6:**
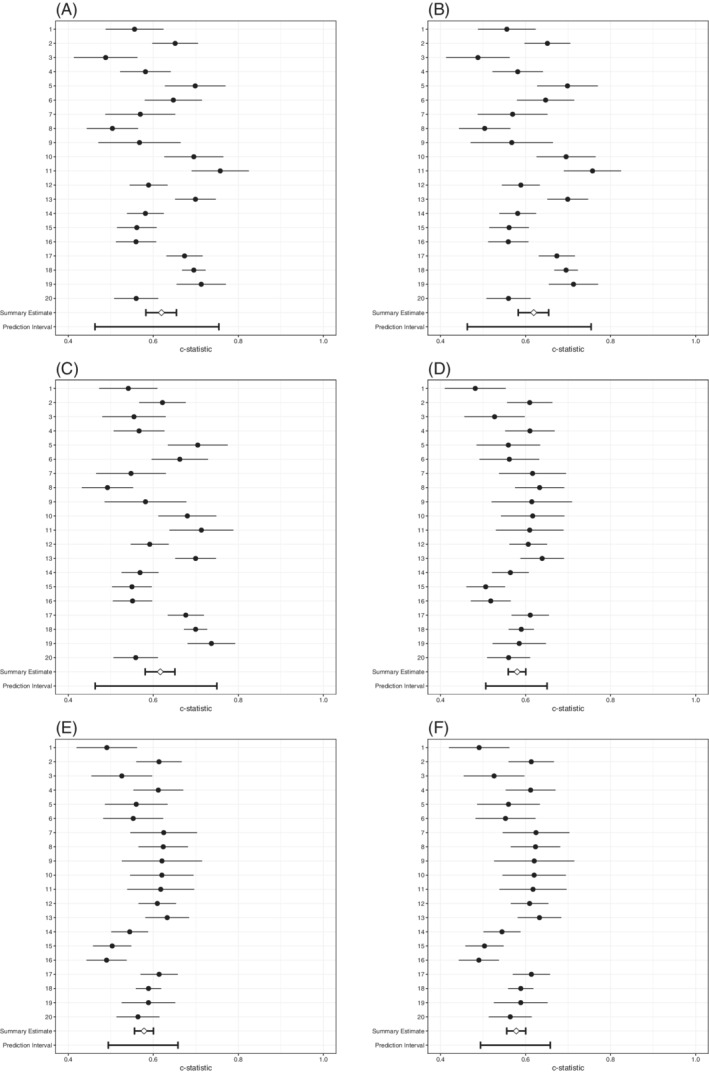
Forest plots of SIECV estimates of c‐statistics of six strategies for predicting AF

The *A* ^M^, $A^{{\text{RE}}_{1}}$, and $A^{{\text{RE}}_{1/2}}$ strategies attained a somewhat better discrimination with c‐statistics of 0.62 (95% CI: 0.58 to 0.65) than the other strategies, that all attained c‐statistics of 0.58 (95% CI: 0.56 to 0.60), respectively (Table 10, Figure 6). There was considerable heterogeneity in the c‐statistics for all strategies. The discrimination was worse than random (c‐statistic < 0.50) in at least one cluster for each of the strategies. Indeed, the approximate 95% prediction interval shows it is most likely that this will occur in a new cluster for the *A* ^M^ and $A^{{\text{RE}}_{1}}$ strategies.

For all of the considered strategies the SIECV shows that although calibration was adequate on average and that overall discrimination was modest, it is not very likely that any of the developed models will perform well at predicting AF in new clusters without local updating. The effects of the included predictors vary substantially across clusters and hence heterogeneity of performance could not be resolved by considering nonlinear terms. The used for these analyses can be found on Github (https://github.com/VMTdeJong/SIECV‐AF).

## 6 GENERAL DISCUSSION

We proposed a methodology for improving the generalizabilty of prediction models developed across different settings and populations when individual participant data from multiple studies or electronic health records are available. Our methodology is aimed at situations where the goal is to produce a single model that can globally be used across a wide variety of settings without having to update or recalibrate predictor effects (although it still allows intercepts to be updated). This methodology leverages the information from multiple clusters (eg, studies) by iteratively using all but one cluster for model development and assessing performance in the remainder.

The overall predictive performance and its variation across clusters can then be used to quantify a model's generalizability across clusters, and to inform the selection of predictors. As we have demonstrated in our motivating examples, the selection of predictors based on the proposed aggregated loss functions can lead to differing model specifications. Each model specification may perform differently in predicting the outcome for individuals in differing clusters, leading to differing average and heterogeneity of calibration and discrimination. Trade‐offs may occur between discrimination and calibration as well as between average and heterogeneity of performance. These may be quantified in the SIECV algorithm during model development, by which the need for extensive or local model updating may be assessed immediately. For instance, before validation in the hold‐out cluster, the intercept may be updated, which will inform the researcher on the generalizability of performance of the model after local updates.

Although it remains unlikely that SIECV can completely resolve the need for an intercept update, it may reduce the necessity of the local tailoring of prediction models (particularly with respect to the calibration slope), which is highly prevalent in the implementation of today's prediction models. In particular, we aimed to reduce the need for re‐estimation of individual predictor effects. Evidently, the potential impact of SIECV strongly depends on the availability of patient‐level covariates that may explain heterogeneity in predictive associations.

We discussed a variety of aggregated loss functions for quantifying the generalizability of a developed model. These ranged from producing an average performance estimate to quantifying its dispersion across clusters. Our framework also allows for assessing average and heterogeneity of performance across data sets (clusters) through meta‐analysis, and formalizes the predictor function selection by both of these simultaneously. This requires specifying the relative importance of average performance and the heterogeneity thereof on beforehand. For the meta‐analysis strategy, this either requires the prespecification of λ, or the finding of an optimal value for λ through a resampling method. When applied in the SIECV model development process, these measures lead to different model specifications with different average performance and heterogeneity thereof, as each places different importance thereon. Therefore, the researcher will have to choose whether to optimize average calibration (calibration‐in‐the‐large close to 0, slope close to 1) and discrimination (high c‐statistic, positive and/or negative predictive value for its clinical issue), or the heterogeneity of one or both of these across clusters.

### 6.1 Limitations and future directions

Our main focus was on informing the selection of predictors in the analysis of multiple studies or EHR, and not on the estimation of corresponding predictor effects. Hence, the impact of our methodology may be limited in cases where the number of available predictors is low or when available predictors are not related to heterogeneity in predictor‐outcome associations. Though, even in cases where we have few predictors, it may still prove worthwhile to consider nonlinear terms and interaction effects by the use of SIECV.

Improvements in one prediction model performance measure may come at the cost of those in another measure. For instance, an improvement in calibration may result in a deterioration in discrimination. In our motivating example, the predictor d‐dimer had a large predictive effect, and substantially contributed to discrimination performance. However, d‐dimer was a dichotomized predictor and was measured using different methods across clusters.^58^, ^79^ This resulted in substantial heterogeneity in its regression coefficients and in heterogeneity of its diagnostic accuracy (see Reference 80).

Differences in predictor distributions will have an impact on the discrimination of an estimated model:^31^ in fact, the c‐statistic is directly related to the SD of the linear predictor.^81^ Differences in predictor distributions should not have any impact on the calibration,^31^ as long as all predictors have been measured and included (with the right functional form) in the prediction model, and all of the regression coefficients are correct and there is no heterogeneity in the true regression coefficients. However, differences in distributions of unmeasured predictors will have an impact on the model's calibration. If these predictors are correlated with measured predictors, this will appear as miscalibration of the linear predictor (ie, the calibration slope) and possibly the intercept. Including this predictor in the prediction model—if it were measured—would then remove this miscalibration, thereby improving performance. If the unmeasured predictor is not related to any included predictors, the prediction model's miscalibration will appear solely as miscalibration of the intercept or calibration‐in‐the‐large. In other words, if the distribution of a predictor changes, the predicted incidence of the outcome should change accordingly. If that predictor is not measured it will not affect the predictions, which leads to miscalibration.

Imprecise regression coefficients may impact each aspect of performance. If all predictors' k coefficients are too large for the validation sample, the calibration slope will be <1, but discrimination will be unaffected. If some are too large and some too small, the discrimination will also be deteriorated. If the model's intercept is incorrect, or if it cannot account for differences in predictor effects, the calibration‐in‐the‐large will be affected, but not the discrimination.

To gain an understanding of the possible values for performance the prediction model may have in a new sample, the use of benchmark values has been proposed.^31^, ^82^ Most importantly, the case‐mix corrected c‐statistic disentangles the effects of case‐mix and incorrect coefficients between clusters. It assumes the predictor coefficients are valid and adjusts for the case‐mix of the validation sample.^31^ An alternative approach is to quantify the relatedness of the different clusters using a membership model.^14^

In this article, we have focused on improving the generalizability of a model's predictive performance through the addition or removal of predictors or functions of predictors. However, this may lead to instability in the estimation process and overfitting of the predictor coefficients if not enough data are available or the outcome is too rare. We stress that the proposed methodology is only likely to lead to stable estimates for the predictor effects when large data sets are available. A future step may be to incorporate heterogeneity of performance into the estimation process. For instance, in penalized maximum likelihood estimation a penalty for heterogeneity of predictor coefficients or predictive performance across clusters could be applied. Such a penalty could readily produce more generalizable models, without the need for a stepwise selection process.

We have not performed a simulation study to compare the aggregated loss functions. Such an endeavor might not be fruitful, as it should be obvious that each of these measures for predictor selection would lead to different estimates of both average and heterogeneity of performance, that is, there is no gold standard. As each of the aggregated loss functions serve a different goal, it is unclear what the evaluation criteria in such a simulation study would be. Nevertheless, it may still be helpful to assess under what circumstances certain generalizability measures may be preferred.

Whereas methodology has been developed on dealing with missing data in model validation in general (eg, see Reference 83), more guidance is needed for application to SIECV. In general, the use of multilevel imputation methods have been recommended in IPD‐MA and in the analysis of other types of clustered data.^76^, ^84^, ^85^, ^86^ These methods can account for variables that are sporadically missing within one or more clusters, but also impute variables that are not measured in certain clusters.^85^ The adoption of multilevel imputation methods therefore seems paramount when adopting SIECV in data sets with missing values. Further, in multiple imputation it is imperative that the imputation model includes the all of the variables used in the analysis model, including the outcome, as well as any other predictive variables.^87^ For this reason, we recommend to include all candidate predictors of SIECV in the imputation model, and to allow for random effects for all of these. Recently, several authors evaluated approaches for implementing multiple imputation in an internal validation procedure. Notably, Schomaker and Heumann showed that the bootstrap and multiple imputation can be applied in sequence to obtain valid SEs, and give recommendations on its implementation.^88^ Further, Wahl et al. recommend that the multiple imputation procedure is performed within the development and validation sets separately, for each split in a k‐fold cross‐validation,^89^ which could readily be extended to IECV. Extension to SIECV, however, would require either an appropriate synthesis of performance estimates across imputed data sets within studies before calculation of the aggregate loss function and predictor selection, or an appropriate synthesis of selected predictors in different imputed data sets. For the latter, a voting strategy may be applied.^90^, ^91^ When data are missing sporadically and a two‐stage meta‐analysis approach is taken, the imputation procedure might be performed within clusters before applying SIECV. A formal comparison of these strategies is still needed.

## 7 CONCLUSION

The IECV methodology for model validation can inform the researcher on the need for updating a prediction model to adapt it to particular populations and settings, when IPD from multiple clusters or studies are available. Our SIECV methodology extends this framework to quantify and reduce the impact of any heterogeneity on prediction model performance. This can inform the researcher and may reduce the need for tailoring the prediction model to specific populations and settings.

## Supporting information


**Data S1.** Online supporting information A and B

## Data Availability

The DVT data that support the findings of this study are not publicly available, according to the conditions determined by the authors of the DVT studies, but are available on request from G.J. Geersing, by e‐mailing G.J.Geersing@umcutrecht.nl. The atrial fibrillation data that support the findings of this study are openly available in The Comprehensive R Archive Network at https://cran.r‐project.org/,^30^ in R package micemd.^92^
