# Supplementary material for: Developing more generalizable prediction models from pooled studies and large clustered data sets
Source: Stat Med. 2021 May 5;40(15):3533–59. doi: 10.1002/sim.8981 (PMC8252590; doi:10.1002/sim.8981)
Supplement: Supplementary file 1 — Data S1. Online supporting information A and B [file SIM-40-3533-s001.docx]

# Online supporting information A: simulation study

In this simulation study, we investigate the differences in 1) selected predictors, 2) out-of-sample average predictive performance, and 3) heterogeneity of out-of-sample predictive performance for two modeling strategies that use stepwise internal-external cross-validation (SIECV): forwards selection and backwards elimination.

**Methods**

We simulate 7 binary and 2 continuous variables, for which we estimated the parameters that we used to generate data as follows in the DVT data:

- We estimated the prevalence of each binary predictor in each cluster separately.
- We standardized the continuous predictors within each cluster.
- We estimated the average covariance of the predictors in each cluster separately.
- We estimated the summary coefficient value of each of these predictors in a two-stage meta-analyses of logistic models, where the predictors were included with linear effects.

Subsequently, we used the following procedure to generate cluster-specific parameters in the simulation:

- For two predictors, the predictive value was minimal and the p-value greater than .50, and so we set their coefficients to 0.
- The resulting summary coefficients were used as the true mean effects in the simulation.
- The standard deviations (tau) estimated for each coefficient in the two-stage meta-analyses were used to sample cluster-specific effects for each of the variables.
- For each predictor separately, we sampled with replacement from the estimates of the prevalence in the different clusters.

To generate the individual participant data for 11 clusters:

- We sampled predictor data for all predictors from a multivariate normal distribution, using the estimated average covariance per cluster.
- We generated binary predictor data by dichotomizing the normal predictors at the cutoff that yielded the corresponding prevalence.

The resulting samples for each cluster and coefficients for each cluster were then used to predict the probability of the outcome for each observation in each cluster, and subsequently the outcomes were sampled from binomial distributions.

To compare forwards selection and backwards elimination, we applied the SIECV algorithm twice, once with forward selection and once with backwards elimination of predictor variables. Both were estimated with a binomial logistic model, used the MSE (i.e. Brier score) as cross-validation loss function within clusters and used A^MA^½ as aggregated cross-validation loss function.

We repeat the entire sampling procedure to generate a validation set of 11 clusters, in which we validate both models, in terms of

1. average of the MSE of the predicted outcomes in the validation sets, and
2. heterogeneity estimates (tau^2) of the MSE of the predicted outcomes in the different validation sets, resulting from random effects meta-analysis estimated with REML.

Subsequently, we performed a second simulation, which was the same as the first with the exception that three interaction effects and a nonlinear effect were included in the full model, similar to the model formula in the DVT applied example. The R code for both simulations is available online: https://github.com/VMTdeJong/SIECV-sim-DVT

**Results**

In these simulations, we found that the models selected the exact same predictors in 990 out of 1000 the simulation repetitions. With both strategies an average of 6.0 predictors were selected. The resulting out-of-sample performance (in terms of average MSE of predicted outcomes) was not statistically different t(999) = 0.25, p = .81, and not clinically significant, mean difference in MSE = 3.3e-06. In terms of heterogeneity of performance (tau^2 of MSE of predicted outcomes) there was also no statistically significant difference t(999) = -1.18, df = 999, p = 0.24, and no clinically significant difference, mean difference in tau^2 = -9.5e-07.

In the second simulation, where we amended the simulation such that three interaction effects and a nonlinear effect were included in the full model, similar to the model formula in the DVT applied example, in only 216 out of 1000 simulation repetitions the two strategies produced the exact same model. On average, the forward selection strategy selected 7.0 predictors and the backwards elimination strategy selected 8.0. But still the average out-of-sample performance remained statistically insignificant t(999) = -0.11, p = 0.91 and clinically insignificant, mean difference in MSE = -1.45e-06. Also, we find no statistically significant difference in heterogeneity of out-of-sample performance, t(999) = -1.09, df = 999, p-value = 0.28, and no clinically significant difference, mean difference in tau^2 = -9.21e-07.

**Conclusion**

These results show that in this realistic clinical data set, there is no meaningful difference between the performance of the forwards selection and backwards elimination approaches to SIECV. Of course, this is only one example and further evaluations would be welcome in the future.

# Online supporting information B: missing data

**Proportions of missing data in Atrial Fibrillation clusters**

| \|  \| gender \| bmi \| age \| sbp \| dbp \| hr \| lvef \| bnp \| afib \| \| --- \| --- \| --- \| --- \| --- \| --- \| --- \| --- \| --- \| --- \| \| 1 \| 0.00 \| 0.36 \| 0.02 \| 0.01 \| 0.02 \| 0.03 \| 0.00 \| 0.57 \| 0.00 \| \| 2 \| 0.00 \| 0.19 \| 0.05 \| 0.02 \| 0.03 \| 0.01 \| 0.00 \| 0.10 \| 0.00 \| \| 4 \| 0.00 \| 0.02 \| 0.01 \| 0.01 \| 0.01 \| 0.02 \| 0.00 \| 0.04 \| 0.42 \| \| 6 \| 0.00 \| 1.00 \| 0.03 \| 1.00 \| 1.00 \| 0.01 \| 0.00 \| 1.00 \| 0.00 \| \| 7 \| 0.00 \| 0.00 \| 0.05 \| 0.01 \| 0.02 \| 0.01 \| 0.00 \| 0.02 \| 0.00 \| \| 8 \| 0.00 \| 0.44 \| 0.07 \| 0.16 \| 0.16 \| 0.19 \| 0.00 \| 0.12 \| 0.22 \| \| 9 \| 0.00 \| 0.01 \| 0.04 \| 0.00 \| 0.00 \| 0.00 \| 0.00 \| 0.00 \| 0.00 \| \| 12 \| 0.00 \| 0.27 \| 0.03 \| 0.00 \| 0.00 \| 0.01 \| 0.00 \| 1.00 \| 0.00 \| \| 14 \| 0.00 \| 0.77 \| 0.03 \| 0.01 \| 0.01 \| 0.02 \| 0.00 \| 1.00 \| 0.00 \| \| 15 \| 0.00 \| 0.13 \| 0.04 \| 0.02 \| 0.02 \| 0.02 \| 0.00 \| 0.82 \| 0.00 \| \| 16 \| 0.00 \| 0.00 \| 0.04 \| 0.00 \| 0.00 \| 0.00 \| 0.00 \| 1.00 \| 0.00 \| \| 18 \| 0.00 \| 0.19 \| 0.03 \| 0.01 \| 0.01 \| 0.00 \| 0.00 \| 0.92 \| 0.00 \| \| 19 \| 0.00 \| 0.07 \| 0.02 \| 0.00 \| 0.00 \| 0.00 \| 0.00 \| 0.99 \| 0.00 \| \| 21 \| 0.00 \| 0.10 \| 0.06 \| 0.00 \| 0.00 \| 0.00 \| 0.00 \| 0.97 \| 0.00 \| \| 22 \| 0.00 \| 0.24 \| 0.03 \| 0.02 \| 0.02 \| 0.02 \| 0.00 \| 0.73 \| 0.02 \| \| 23 \| 0.00 \| 0.02 \| 0.02 \| 0.00 \| 0.00 \| 0.00 \| 0.00 \| 0.87 \| 0.00 \| \| 24 \| 0.00 \| 0.04 \| 0.03 \| 0.00 \| 0.00 \| 0.00 \| 0.00 \| 0.81 \| 0.00 \| \| 25 \| 0.00 \| 0.01 \| 0.04 \| 0.01 \| 0.01 \| 0.01 \| 0.00 \| 1.00 \| 0.01 \| \| 26 \| 0.00 \| 0.11 \| 0.05 \| 0.00 \| 0.00 \| 0.00 \| 0.00 \| 0.66 \| 0.00 \| \| 27 \| 0.00 \| 0.11 \| 0.03 \| 0.00 \| 0.00 \| 0.00 \| 0.00 \| 0.79 \| 0.00 \| |  |  |  |  |  |  |  |  |  |
| --- | --- | --- | --- | --- | --- | --- | --- | --- | --- | --- | --- | --- | --- | --- | --- | --- | --- | --- | --- | --- | --- | --- | --- | --- | --- | --- | --- | --- | --- | --- | --- | --- | --- | --- | --- | --- | --- | --- | --- | --- | --- | --- | --- | --- | --- | --- | --- | --- | --- | --- | --- | --- | --- | --- | --- | --- | --- | --- | --- | --- | --- | --- | --- | --- | --- | --- | --- | --- | --- | --- | --- | --- | --- | --- | --- | --- | --- | --- | --- | --- | --- | --- | --- | --- | --- | --- | --- | --- | --- | --- | --- | --- | --- | --- | --- | --- | --- | --- | --- | --- | --- | --- | --- | --- | --- | --- | --- | --- | --- | --- | --- | --- | --- | --- | --- | --- | --- | --- | --- | --- | --- | --- | --- | --- | --- | --- | --- | --- | --- | --- | --- | --- | --- | --- | --- | --- | --- | --- | --- | --- | --- | --- | --- | --- | --- | --- | --- | --- | --- | --- | --- | --- | --- | --- | --- | --- | --- | --- | --- | --- | --- | --- | --- | --- | --- | --- | --- | --- | --- | --- | --- | --- | --- | --- | --- | --- | --- | --- | --- | --- | --- | --- | --- | --- | --- | --- | --- | --- | --- | --- | --- | --- | --- | --- | --- | --- | --- | --- | --- | --- | --- | --- | --- | --- | --- | --- | --- | --- | --- | --- | --- | --- | --- | --- | --- | --- | --- | --- | --- |

| **Proportions of missing data in DVT clusters**   \|  \| sex \| age \| durat \| malign \| par \| surg \| tend \| leg \| calfdif3 \| pit \| vein \| altdiagn \| notraum \| histdvt \| coag \| ddimdich \| typdim \| dvt \| wells \| \| --- \| --- \| --- \| --- \| --- \| --- \| --- \| --- \| --- \| --- \| --- \| --- \| --- \| --- \| --- \| --- \| --- \| --- \| --- \| --- \| \| 1 \| 0.00 \| 0.00 \| 0.00 \| 0.00 \| 0.00 \| 0.00 \| 0.00 \| 0.00 \| 0.00 \| 0.00 \| 0.00 \| 0.00 \| 0.00 \| 0.00 \| 0.00 \| 0.00 \| 0.00 \| 0.00 \| 0.00 \| \| 2 \| 0.00 \| 0.00 \| 1.00 \| 0.00 \| 0.00 \| 0.00 \| 0.00 \| 0.00 \| 0.00 \| 0.00 \| 0.00 \| 0.00 \| 1.00 \| 0.00 \| 1.00 \| 0.00 \| 0.00 \| 0.00 \| 0.00 \| \| 3 \| 0.00 \| 0.00 \| 0.00 \| 0.00 \| 0.00 \| 0.00 \| 0.00 \| 0.00 \| 0.00 \| 0.00 \| 0.00 \| 0.00 \| 0.00 \| 0.00 \| 1.00 \| 0.00 \| 0.00 \| 0.00 \| 0.00 \| \| 4 \| 0.00 \| 0.00 \| 0.00 \| 0.00 \| 0.00 \| 0.00 \| 0.00 \| 0.00 \| 0.00 \| 0.00 \| 0.00 \| 0.00 \| 0.00 \| 0.00 \| 0.00 \| 0.00 \| 0.00 \| 0.00 \| 0.00 \| \| 5 \| 0.00 \| 0.00 \| 0.00 \| 0.00 \| 0.00 \| 0.00 \| 0.00 \| 0.00 \| 0.00 \| 0.00 \| 0.00 \| 0.00 \| 0.00 \| 0.00 \| 0.00 \| 0.00 \| 0.00 \| 0.00 \| 0.00 \| \| 6 \| 0.00 \| 0.00 \| 0.00 \| 0.00 \| 0.00 \| 0.00 \| 0.00 \| 0.00 \| 0.00 \| 0.00 \| 0.00 \| 0.00 \| 0.00 \| 0.00 \| 0.00 \| 0.00 \| 0.00 \| 0.00 \| 0.00 \| \| 7 \| 0.00 \| 0.00 \| 0.00 \| 0.00 \| 0.00 \| 0.00 \| 0.00 \| 0.00 \| 0.00 \| 0.00 \| 0.00 \| 0.00 \| 1.00 \| 0.00 \| 1.00 \| 1.00 \| 1.00 \| 0.00 \| 0.00 \| \| 8 \| 0.00 \| 0.00 \| 1.00 \| 0.00 \| 0.00 \| 0.00 \| 0.00 \| 0.00 \| 0.00 \| 0.00 \| 0.00 \| 0.00 \| 1.00 \| 0.00 \| 1.00 \| 0.00 \| 0.00 \| 0.00 \| 0.00 \| \| 9 \| 0.00 \| 0.00 \| 0.00 \| 0.00 \| 0.00 \| 0.00 \| 0.00 \| 0.00 \| 0.00 \| 0.00 \| 0.00 \| 0.00 \| 0.00 \| 0.00 \| 1.00 \| 0.00 \| 0.00 \| 0.00 \| 0.00 \| \| 10 \| 0.00 \| 0.00 \| 1.00 \| 0.00 \| 0.00 \| 0.00 \| 0.00 \| 0.00 \| 0.00 \| 0.00 \| 0.00 \| 0.00 \| 1.00 \| 0.00 \| 0.00 \| 1.00 \| 1.00 \| 0.00 \| 0.00 \| \| 11 \| 0.00 \| 0.00 \| 0.00 \| 0.00 \| 0.00 \| 0.00 \| 0.00 \| 0.00 \| 0.00 \| 0.00 \| 0.00 \| 0.00 \| 0.00 \| 1.00 \| 0.00 \| 0.00 \| 0.00 \| 0.00 \| 0.00 \| |
| --- | --- | --- | --- | --- | --- | --- | --- | --- | --- | --- | --- | --- | --- | --- | --- | --- | --- | --- | --- | --- | --- | --- | --- | --- | --- | --- | --- | --- | --- | --- | --- | --- | --- | --- | --- | --- | --- | --- | --- | --- | --- | --- | --- | --- | --- | --- | --- | --- | --- | --- | --- | --- | --- | --- | --- | --- | --- | --- | --- | --- | --- | --- | --- | --- | --- | --- | --- | --- | --- | --- | --- | --- | --- | --- | --- | --- | --- | --- | --- | --- | --- | --- | --- | --- | --- | --- | --- | --- | --- | --- | --- | --- | --- | --- | --- | --- | --- | --- | --- | --- | --- | --- | --- | --- | --- | --- | --- | --- | --- | --- | --- | --- | --- | --- | --- | --- | --- | --- | --- | --- | --- | --- | --- | --- | --- | --- | --- | --- | --- | --- | --- | --- | --- | --- | --- | --- | --- | --- | --- | --- | --- | --- | --- | --- | --- | --- | --- | --- | --- | --- | --- | --- | --- | --- | --- | --- | --- | --- | --- | --- | --- | --- | --- | --- | --- | --- | --- | --- | --- | --- | --- | --- | --- | --- | --- | --- | --- | --- | --- | --- | --- | --- | --- | --- | --- | --- | --- | --- | --- | --- | --- | --- | --- | --- | --- | --- | --- | --- | --- | --- | --- | --- | --- | --- | --- | --- | --- | --- | --- | --- | --- | --- | --- | --- | --- | --- | --- | --- | --- | --- | --- | --- | --- | --- | --- | --- | --- | --- | --- | --- | --- | --- | --- | --- | --- | --- | --- | --- | --- | --- |
